# Fabrication of Low-Fouling Surfaces on Alkyne-Functionalized Poly-(p-xylylenes) Using Click Chemistry

**DOI:** 10.3390/polym14020225

**Published:** 2022-01-06

**Authors:** Pei-Ju Chen, Hsien-Yeh Chen, Wei-Bor Tsai

**Affiliations:** Department of Chemical Engineering, National Taiwan University, 1, Roosevelt Rd., Sec. 4, Taipei 10617, Taiwan; r02524057@ntu.edu.tw (P.-J.C.); hsychen@ntu.edu.tw (H.-Y.C.)

**Keywords:** chemical vapor deposition, poly-(p-xylylene), anti-fouling, polysulfobetaine, thermo-responsive, click chemistry

## Abstract

A facial, versatile, and universal method that breaks the substrate limits is desirable for antifouling treatment. Thin films of functional poly-p-xylylenes (PPX) that are deposited using chemical vapor deposition (CVD) provide a powerful platform for surface immobilization of molecules. In this study, we prepared an alkyne-functionalized PPX coating on which poly (sulfobetaine methacrylate-co-Az) could be conjugated via click chemistry. We found that the conjugated polymers were very stable and inhibited cell adhesion and protein adsorption effectively. The same conjugation strategy could also be applied to conjugate azide-containing poly (ethylene glycol) and poly (NIPAAm). The results indicate that our method provides a simple and robust tool for fabricating antifouling surfaces on a wide range of substrates using CVD technology of functionalized poly (p-xylylenes) for biosensor, diagnostics, immunoassay, and other biomaterial applications.

## 1. Introduction

When biomaterials are in contact with the biological environment, nonspecific protein adsorption and cell adhesion occur unavoidably, often leading to malfunction of biomedical devices. Antifouling surfaces that resist nonspecific protein adsorption and cell adhesion are demanded to reduce adverse biological responses [1]. An important feature of antifouling surfaces is their ability to bind water molecules tightly. Therefore, many hydrophilic polymers, such as poly (ethylene glycol), polyglycerol, and poly (zwitterions), have been conjugated to biomaterials for antifouling [2,3,4,5,6,7,8,9,10]. For example, poly (zwitterions) with high water binding capacity via electrostatic interactions have been shown to be excellent antifouling materials [5,6,8,9,11,12]. Therefore, many research efforts have focused on immobilization of antifouling polymers on biomaterial surfaces to reduce unwanted biological interactions.

Approaches for the surface coupling of antifouling polymers could be classified into two main categories, ‘graft-to’ and ‘graft-from’ via physical or chemical conjugation [12]. Covalent conjugation of antifouling polymers is more desirable because of its long-term stability. However, many methods for the chemical immobilization of antifouling polymers on biomaterials surfaces are tedious, time-consuming, and substrate-dependent. Therefore, a facial, versatile, and universal method that breaks substrate limits is welcome for antifouling treatment.

We previously developed a photoreactive carboxybetaine-based polymer containing azidoaniline, which forms photo-crosslinking to several substrates via UV irradiation [13]. When the phenylazide groups of photoreactive polymers are exposed to UV irradiation, they are converted into reactive nitrenes that can be inserted into the C–H bonds of the surrounding macromolecules and surfaces [14] so as to immobilize the carboxybetaine-based polymer on a substrate to create a versatile low-fouling platform that reduces protein adsorption/cell adhesion. This mechanism has been used for the crosslinking of polyelectrolyte multilayer films and scaffolds for biomedical applications [6,7,15,16].

In addition to the photo-initiated reaction, azides could form chemical bonds with alkynes through click chemistry. Click chemistry stands for a family of powerful, fast, and reliable chemical reactions that generate substances efficiently by joining small units together [17]. The features of click chemistry include modular, wide-ranging in scope, with very high chemical yields, relatively insensitive to solvents and pH, and stereospecific. The most widely used click reaction is Huisgen 1,3-dipolar cycloaddition between azides and alkynes in the presence of a copper (I) catalyst, often referred to as copper (I)-catalyzed azide-alkyne cycloaddition (CuAAC) [18,19]. Thus, azide-containing polycarboxybetaine could be conjugated to an alkyne-functionalized surface via click chemistry.

Thin films of functional poly-*p*-xylylenes (PPX) that are coated by chemical vapor deposition (CVD) provide a powerful platform for surface immobilization of biomolecules [20]. This technique possesses several unique features, such as applicability to a wide range of materials, including polymers, silicon, glass and ceramics, ultrathin coating (20–100 nm), solvent-free environment, absence of cytotoxic initiators and plasticizers, and no limit in substrate shapes [20,21,22]. Therefore, the PPX coating is especially suitable for the surface modification of biomedical devices [23]. A wide range of functionalized poly-p-xylyenes with different chemical groups could be used for the creation of functional PPX coatings, which serve as excellent platforms for the covalent immobilization of molecules such as proteins, sugars, or polymers [24]. For effective conjugation of antifouling polymers, we would like to choose a fast and specific reaction. Nandivada et al. developed PPX films containing alkene groups, which could undergo a click reaction with azide-containing molecules [25]. The alkyne-PPX CVD coating provides an excellent platform for conjugation of biomolecules via the surface-directed CuAAC click reaction.

In this study, we applied this alkyne-containing platform for conjugation of a zwitterionic polymer, poly (sulfobetaine methacrylate) (pSBMA), via the CuAAC click reaction. pSBMA has been shown to greatly reduce nonspecific protein adsorption and cell adhesion [6,11,26]. Surface-immobilized pSBMA has been shown to possess a super-low fouling property that adsorbs undetectable protein adsorption (<0.3 ng cm^−2^) from fibrinogen solution [26]. Here, we synthesized an azide-containing pSBMA that was then conjugated to the alkyne-PPX surface via the CuAAC. The antifouling properties of the pSBMA coating were evaluated by attachment of proteins, L929 cells, and platelets. Furthermore, we also demonstrated that other polymers such as poly (ethylene glycol methacrylate) (poly-PEGMA) and poly (N-isopropylacrylamide) (poly-NIPAAm) could also be conjugated on a surface via the same approach to provide specific surface functions.

## 2. Materials and Methods

### 2.1. Synthesis and Characterization of N-(4-Azidophenyl) Methacrylamide (AzMA)

**Scheme 1:**
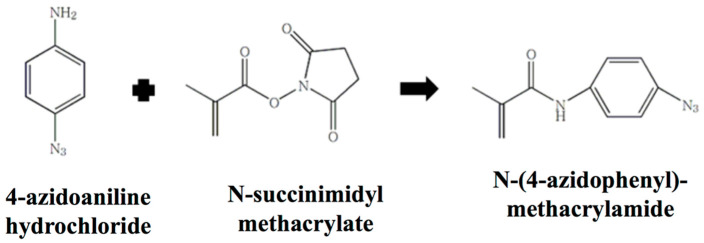
Synthesis of N-(4-azidophenyl)-methacrylamide (AzMA).

The synthetic scheme of N-(4-azidophenyl)methacrylamide is illustrated in Scheme 1, according to a previous procedure [27]. Briefly, 153.2 mg (0.9 mmol) 4-azidoaniline hydrochloride (Cat. No. 359556, Sigma-Aldrich, St. Louis, MO, USA) and 159 mg (1.5 mmol) sodium carbonate were dissolved in 35 mL of deionized water in the dark. 274.14 mg (1.5 mmol) N-succinimidyl methacrylate (Cat. No. 38862-25-8, Tokyo Chemical Industry, Japan) was dissolved in 3 mL of dioxane and then added to the 4-azidoaniline hydrochloride solution. The mixture was allowed to react at room temperature for 6 h in the dark under constant stirring. The product, *N*-(4-azidophenyl)methacrylamide (AzMA), was precipitated, filtrated, washed with deionized water, and then dried. The product was identified by ^1^H NMR (D-methanol, 500 MHz). The characteristic peaks of the hydrogen in the aromatic ring appear at 7.0 and 7.6 ppm (Supplementary Figure S1).

### 2.2. Synthesis and Characterization of Azide-Containing Sulfobetaine Polymers

Azide-containing copolymers were synthesized by copolymerization of AzMA and a monomer including sulfobetaine methacrylate, PEG methacrylate, or *N*-isopropylacrylamide. For the synthesis of poly (sulfobetaine methacrylate-co-*N*-(4-azidophenyl)methacrylamide), 700 mg (2.51 mmol) sulfobetaine methacrylate (SBMA, Cat. No. S059-0203, Hopax, Taiwan), 26.26 mg (0.13 mmol) AzMA, and 3.7 mg (0.0132 mmol) 4,4′-azobis (4-cyanovaleric acid) (Cat. No. 11590, Sigma-Aldrich, St. Louis, MO, USA) were dissolved in methanol and then purged with N_2_ for 1 h, followed by continuous stirring at 70 °C for 12 h. The product was dialyzed against deionized water and then freeze-dried. The copolymer is termed pSBAz. The product was characterized by ^1^H NMR in D_2_O (Supplementary Figure S2). The characteristic peaks of the hydrogen atoms were assigned for SBMA: –CH_3_ at 1.0 ppm, –CH_2_ at 1.1-1.9 ppm, O–CH_2_-CH_2_-N at 4.5 and 3.8 ppm, N–CH_2_-CH_2_-CH_2_-S at 3.2, 3.5, 2.3, and 2.9 ppm, and AzMA: aromatic hydrogen at 7.0 and 7.6 ppm.

The synthesis of pPEGAz and pNIPAz is similar to that of pSBAz, except that SBMA is replaced by PEGMA or NIPAAm. The contents of PEGMA (M_n_ 360, Cat. No. 409537, Sigma-Aldrich) and NIPAAm (Cat. No. 415324, Sigma-Aldrich) in the monomer mixtures were 284 mg (2.51 mmol) and 760 mg (2.50 mmol), respectively. The copolymers are termed pPEGAz and pNIPAz, respectively. Their structures were analyzed by ^1^H NMR analysis (Supplementary Figures S3 and S4). The average molecular weights (Mn) of these polymers were 25,000–42,000 measured by gel permeation chromatography with an OH pak SB-803 HQ column (ShodexTM, Showa Denko Inc., Ridgeville, SC, USA).

### 2.3. Conjugation of Azide-Containing Polymers to Alkyne-PPX via Click Chemistry

Alkyne-PPX was deposited on various substrates using CVD polymerization of 4-ethynyl-[2,2]paracyclophane [25]. Alkyne-PPX was deposited on tissue culture polystyrene (TCPS) for the experiments of electron spectroscopy for X-ray photoelectron spectroscopy (XPS) and cell adhesion, on glass slides for water contact angle measurement, and on gold substrates for quantitation of protein adsorption. The polymers were dissolved in deionized water to the desired concentrations (0.1, 1, 5, or 10 mg/mL). Copper (II) sulfate (1 mol%) and sodium ascorbate (5 mol%; Cat. No. S2127, Sigma-Aldrich, St. Louis, MO, USA) were supplemented to accelerate the reaction. The mixture was added on Alkyne-PPX, and then allowed for an 8 h reaction at room temperature. The substrates were then rinsed with deionized water to remove residual reactants and catalysts, and then dried in a 50 °C oven.

### 2.4. Surface Analysis

Surface wettability was evaluated by measuring static water contact angle (WCA) (FTA-125, First Ten Angstroms, Newark, CA, USA). WCAs of at least six 5 μL droplets were measured for each sample. The surface chemical compositions of the substrates were characterized by X-ray photoelectron spectroscopy (XPS) analysis (AXIS Ultra DLD spectrometer, Kratos Analytical Inc., Manchester, UK) with a monochromated Al K—source at a power of 45 W. Each atom specimen was analyzed at the emission angle of 0° as measured from the surface normal. Data were processed using CasaXPS processing software (version 2.3.15, Casa Software Ltd., Teignmouth, UK). Binding energies were referenced to the aliphatic hydrocarbon peak at 285.0 eV.

### 2.5. The Adhesion of L929 Cells and Platelets

Before cell adhesion experiments, substrates were soaked in a 70% ethanol solution for 30 min, followed by rinses with sterilized deionized water.

The mouse fibroblast-like cell line L929 was obtained from the Food Industry Research and Development Institute (Hsinchu, Taiwan). Cell culture medium consisted of 90% essential alpha minimum medium (α-MEM, pH 7.4), supplemented with 0.5% (*v*/*v*) fungizone, 0.25% (*v*/*v*) gentamycin, 3.7 g/L sodium bicarbonate, 0.689% (*v*/*v*) 2-mercaptoehtanol, and 10% (*v*/*v*) fetal bovine serum (FBS). L929 cells were seeded on the substrates at a density of 2 × 10^4^ cells/cm^2^ for 6 h of culture at 37 °C, followed by rinses with PBS. The microscopic cell images were taken under a phase contrast microscope and then the cell numbers were counted from the images, 5 for each sample.

Washed platelets were isolated from fresh blood from healthy human donors according to a previous procedure [28]. Informed consents from the donors were obtained. The samples were immersed in 1% plasma solution at 37 °C for 1 h, rinsed with PBS, and then blocked with 2 wt% bovine serum albumin solution in PBS at 37 °C for 1 h. Before platelet adhesion experiments, the platelet solution was implemented with 1 mM Ca^2+^ and 1 mM Mg^2+^. Platelets were inoculated on the samples at a density of 1.6 × 10^7^ platelets/cm^2^ for 1 h at 37 °C. The unadhered platelets were rinsed away with PBS. The density of platelets adhered was quantified by a lactate dehydrogenase (LDH) activity assay [29].

For cell culture on the pNIPAAm surfaces, C2C12 cells, received from the Food Industry Research and Development Institute of Taiwan, were seeded on the substrates at a density of 5 × 10^4^ cells/cm^2^. After 2 days of culture at 37 °C, the cells became confluent on all substrates. The plates were then placed at room temperature and the detachment of the cell sheets was observed.

### 2.6. Determination of Protein Adsorption Using Quartz Crystal Microbalance (QCM)

The amount of serum protein adsorption on the modified surface of p(SBMA-co-AzMA) was determined using QCM measurement (ANTQ300, ANT Inc., Taipei, Taiwan). Prior to the measurement, PBS was degassed and flowed through a QCM chip (ca. 9 MHz resonance frequency) at a flow rate of 35 µL/min until an equilibrium was reached at which the frequency change was less than 0.01 Hz/s. A total of 150 µL of cell culture medium containing 10% FBS flowed through the chamber at 35 µL/min, followed by a flow of PBS to remove unbound proteins until the frequency reached equilibrium. The amounts of adsorbed proteins were determined from frequency changes, according to the Sauerbrey equation [30].

### 2.7. Statistical Analysis

All data were reported as means ± standard deviation (sample number ≥ 4). Statistical analysis was performed using ANOVA. Probabilities of *p* < 0.05 was considered statistically significant.

## 3. Results and Discussion

### 3.1. Characterization of the Surfaces That Were Conjugated with Azide-Containing Sulfobetaine Polymers

Alkyne-PPX was conjugated with pSBAz by click chemistry for 8 h. Surface chemical compositions were analyzed using XPS. The results indicated the appearance of sulfur and nitrogen atoms after conjugation of Alkyne-PPX with pSBAz (Table 1). We found that the nitrogen and sulfur content increased with increasing concentration of pSBAz from 0.1 to 1 mg/mL. However, no obvious increase in N and S signals was found with increasing concentrations of pSBAz. This is probably because the pSBAz conjugation is monolayer and sulfur and nitrogen atoms are minor elements.

Surface wettability was evaluated using static water contact angle measurement. Conjugation of 0.1 mg/mL pSBAz decreased the water contact angle of PPA-alkyne from 78.4° to 43.4° (Table 1). A further increase in polymer concentrations to 1–10 mg/mL drops water contact angles to −25°, but there were no significant differences between them. The results demonstrated that the conjugation of hydrophilic pSBAz with Alkyne-PPX.

### 3.2. The Adhesion of L929 Cells and Platelets to pSBAz-Modified Surfaces

**Figure 1:**
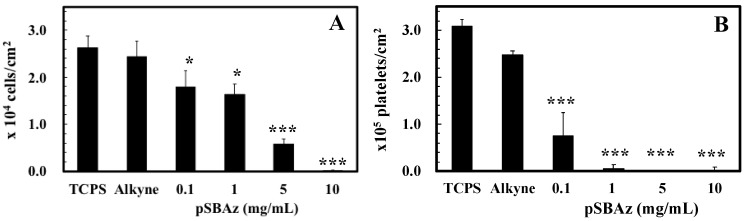
Alkyne-PPX was deposited on tissue culture polystyrene (TCPS) and then conjugated with pSBAz at various concentrations (0.1, 1, 5, and 10 mg/mL). (**A**) 6 h adhesion of L929 cells and (**B**) 1 h adhesion of platelets, on the various substrates. Value = mean ± standard deviation, *n* = 5. *, *** represent *p* < 0.05 and 0.001, respectively, compared to the adhesion of L929 cells or platelets to the corresponding Alkyne.

The conjugation of pSBAz from a 0.1 mg/mL polymer solution significantly decreased the adhesion of L929 cells from 2.44 × 10^4^ cells/cm^2^ on Alkyne-PPX to 1.64 × 10^4^ cells/cm^2^ (*p* < 0.05, Figure 1A). An increase in polymer concentrations further enhanced the cell resistance of the substrates. When the Alkyne-PPX surface was conjugated with a 10 mg/mL polymer solution, cell adhesion was almost completely blocked (~40 cells/cm^2^, *p* < 0.001 vs. Alkyne-PPX). Similarly, platelet adhesion was also greatly depressed on modified surfaces with pSBAz (Figure 1B). However, pSBAz conjugation merely at 1 mg/mL was sufficient to resist more than 99% platelet adhesion on Alkyne-PPX (2.47 × 10^5^ platelet/cm^2^). Platelet adhesion results were consistent with the water contact angle data. Platelet adhesion was almost inhibited on Alkyne-PPX conjugated with 1, 5, or 10 mg/mL of pSBAz, while no differences in WCA were found between these surfaces. However, L929 cell adhesion did not show a similar trend, so the three substrates should have minor differences.

### 3.3. The Adsorption of Serum Proteins to pSBAz-Modified Surfaces

**Figure 2:**
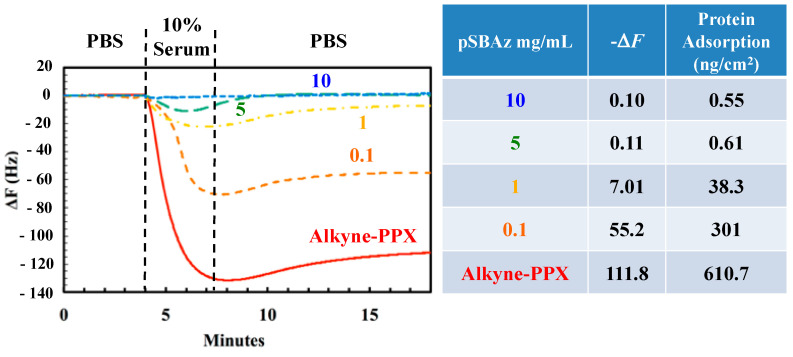
QCM analysis of protein adsorption from 10% fetal bovine serum to piezoelectric quartz disks modified with Alkyne-PPX and pSBAz at 0.1, 1, 5, or 10 mg/mL. 10% FBS flowed through the QCM chamber, followed by flowing PBS to remove unbound proteins until the frequency reached equilibrium. The amounts of adsorbed proteins were determined from frequency changes, according to the Sauerbrey equation.

Since cell adhesion to artificial surfaces is mainly mediated by surface-bound serum cell-adhesion proteins, the adsorption of serum proteins on the substrates was evaluated in order to verify whether the inhibition in cell adhesion is indeed owing to the reduction in protein adsorption from the culture medium. When Alkyne-PPX was immobilized with 0.1 mg/mL of pSBAz, protein adsorption was decreased by 50% from 610.7 to 301 ng/cm^2^ (Figure 2). When the pSBAz concentration was increased to 1 mg/mL, protein adsorption was further reduced to 38.3 ng/cm^2^, respectively. When the pSBAz was increased to 5 and 10 mg/mL, protein adsorption was decreased to a very low level, 0.61, and 0.55 ng/cm^2^, respectively. Our results indicate that conjugation of pSBAz via click chemistry created an ultra-low fouling surface.

### 3.4. The Stability of pSBAz Deposition

Azido-compounds not only form covalent bonds to alkyne groups via the click reaction but also react to neighboring molecules by UV activation [6,16,31]. The deposition of pSBAz via the UV-activation mechanism is easier than that via the click chemistry, because no CVD pretreatment is needed. In this study, we would like to compare the cell-repellent ability and the stability of pSBAz deposition via the two methods.

**Figure 3:**
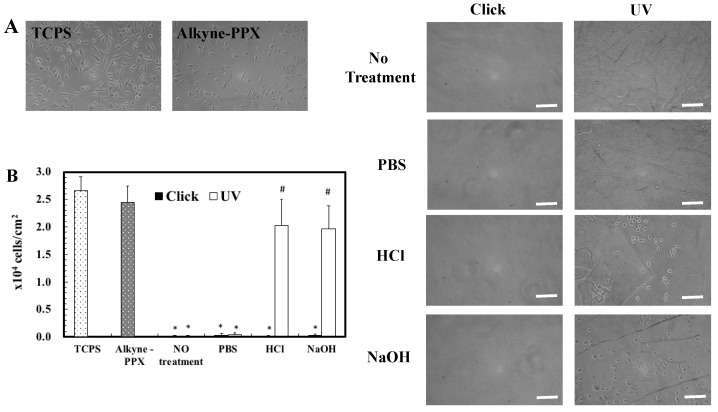
Stability of pSBAz coatings in PBS, 1 N HCl, or 1N NaOH. TCPS was first deposited with Alkyne-PPX, and then cross-linked with pSBAz via click chemistry or UV irradiation. The modified surfaces of pSBAz were incubated in PBS, 1 N HCl, or 1 N NaOH for 24 h. The adhesion of L929 cells was allowed on various surfaces for 6 h. (**A**) Representative microscopic images of cell adhesion on various substrates. Scale bar = 100 μm. (**B**) Quantified data of cell adhesion. Value = mean ± standard deviation, *n* = 5. * Represent *p* < 0.001, compared to the cell adhesion to Alkyne-PPX; # *p* < 0.001, compared to the cell adhesion to the corresponding Click.

While L929 cells adhered well to TCPS and Alkyne-PPX, the deposition of pSBAz via either UV irradiation or the click reaction almost completely inhibits cell adhesion (Figure 3A). However, we found that the deposition of pSBAz via UV irradiation does not look as uniform as that of the “click” surface from the microscopic observation. Both surfaces were stable and remained the cell-resistance after soaking in PBS for 24 h. However, UV-immobilized pSBAz lost the cell-resistant ability after soaking in either 1 N HCl or NaOH for 24 h, indicating that the UV-immobilized pSBAz film is not stable under such harsh conditions. Furthermore, the pSBAz films via UV-initiated crosslinking seemed broken after 1 N HCl or 1 N NaOH treatment (Figure 3A). On the other hand, the pSBAz deposition via the click reaction remained stable under HCl and NaOH incubation, and was still resistant to cell attachment. The quantitative data of cell adhesion indicate that the “click-on” pSBAz retained the anti-cell adhesion property (Figure 3B). Although we previously showed that the UV-crosslinked pSBAz films resisted 1 h incubation of 1 N HCl or 1 N NaOH [13], the film could not survive after long time (24 h) incubation at the strong acidic and alkaline conditions. The results indicate that the immobilization via the click chemistry is more robust that that via UV immobilization.

### 3.5. Conjugation of Other Functional Polymers via Click Reaction

The same conjugation strategy could be also applied to other functional polymers. We here demonstrated the fabrication of poly (ethylene glycol) (PEG) and poly (NIPAAm) surfaces via click chemistry.

**Figure 4:**
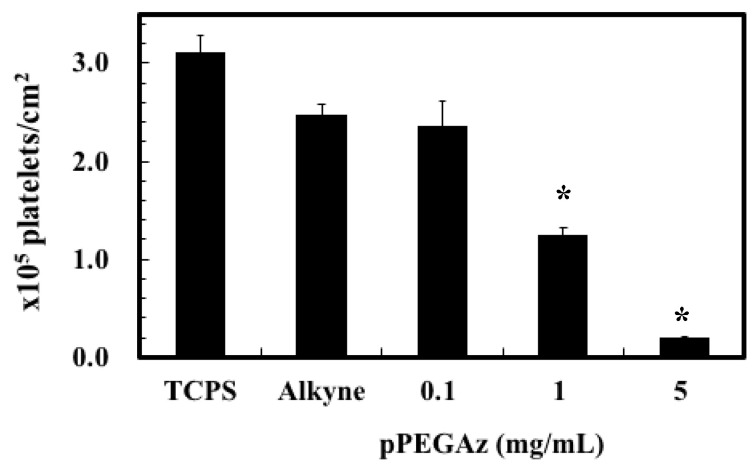
Alkyne-PPX was deposited on TCPS and then conjugated with pPEGAz at various concentrations (0.1, 1, and 5 mg/mL). Platelets were allowed to attach on the various substrates for one hour. Value = mean ± standard deviation, *n* = 5. * *p* < 0.001, compared to the platelet adhesion to Alkyne.

PEG is commonly used as an antifouling material. pPEGAz was conjugated to Alkyne-PPX and showed inhibitory efficacy in platelet adhesion (Figure 4). The conjugation of pPEGAz from a 5 mg/mL solution reduced platelet adhesion to a very low level (0.18 × 10^5^ platelets/cm^2^.), on TCPS and 2.5 × 10^5^ platelets/cm^2^ on Alkyne-PPX (*p* < 0.001). The results indicate that the same method could be applied to PEG conjugation.

**Figure 5:**
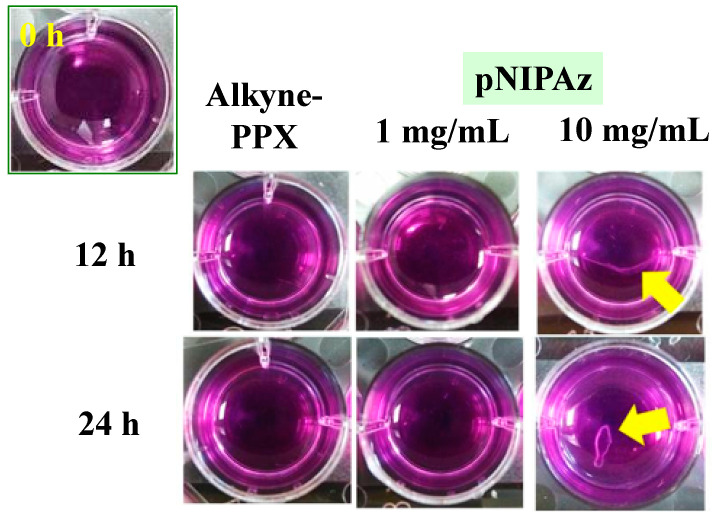
The temperature responsive release of C2C12 cell sheets. Alkyne-PPX was deposited on TCPS and then conjugated with p (NIPAz) at 1 or 10 mg/mL. After cultured for 24 h at 37 °C, the plate was placed at room temperature. The yellow arrows indicate the detached cell sheet.

Next, PNIPAAm was deposited via the click reaction and tested for its cell release property. C2C12 skeletal muscle myoblasts adhered, spread, proliferated, and reached confluence on pNIPAz after 24 h of incubation (Figure 5). No observable morphological and proliferative differences were found in C2C12 cells on the different substrates. After being placed at room temperature for 12 h, the cell layer on 10 mg/mL pNIPAz-modified surface was released from the edge and completely detached from the substrates after 24 h. However, the cell layer on 1 mg/mL pNIPAz-modified surface was not released from the substrates.

## 4. Conclusions

In this study, we demonstrated that via surface deposition of alkyne groups using CVD technology, pSBAz was able to be conjugated to substrates and increased their wettability. pSBAz-modified surfaces resisted the attachment of cells, platelets, and protein. Similarly, other functional polymers containing azides could also be anchored and performed their functions. The results indicate that our method provides a simple and robust tool for fabricating antifouling surfaces on a wide range of substrates using CVD technology of functionalized poly-(p-xylylenes) for biosensor, diagnostics, immunoassay, and other biomaterial applications.

## Figures and Tables

**Table 1:** The atomic compositions and water contact angle (WCA) of Alkyne-PPX and pSBAz conjugated Alkyne-PPX.

pSBAz	Atomic Percentage				WCA
	C	O	N	S	
Alkyne-PPX	97.74	2.25	-	-	78.4° ± 2.4° *
0.1 mg/mL	96.00	2.35	1.23	0.42	44.4° ± 3.2° *
1 mg/mL	95.36	2.29	1.68	0.67	71.4° ± 4.5° *
5 mg/mL	95.74	1.89	1.58	0.83	25.1° ± 2.5° *
10 mg/mL	95.54	2.21	1.48	0.77	26.7° ± 1.8° *

* *p* < 0.001 vs. Alkyne-PPX.

## Data Availability

Not applicable.

## Supplementary Materials

The following are available online at https://www.mdpi.com/article/10.3390/polym14020225/s1, Figure S1: The ^1^H NMR spectrum of acryloyl 4-azobenzene; Figure S2: The ^1^H NMR spectra of p (SBMA) and p (SBMA-co-AzMA); Figure S3: The ^1^H NMR spectrum of poly (PEGMA-co-AzMA); Figure S4: The ^1^H NMR spectrum of poly (PNIPAAm-co-Az).
