# Supplementary material for: Fabrication of Low-Fouling Surfaces on Alkyne-Functionalized Poly-(p-xylylenes) Using Click Chemistry
Source: Polymers (Basel). 2022 Jan 6;14(2):225. doi: 10.3390/polym14020225 (PMC8780154; doi:10.3390/polym14020225)

Supplementary Materials

Figure S1 The ^{1}H NMR spectrum of acryloyl 4-azobenzene

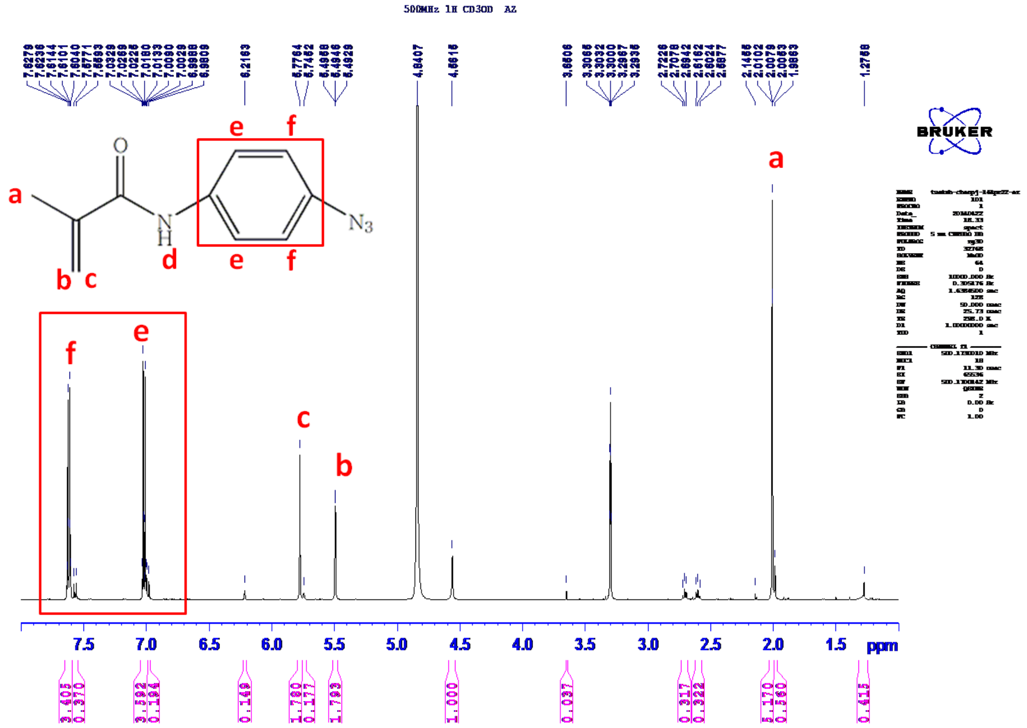

Figure S2 The ^{1}H NMR spectra of p(SBMA) and p(SBMA-co-AzMA)

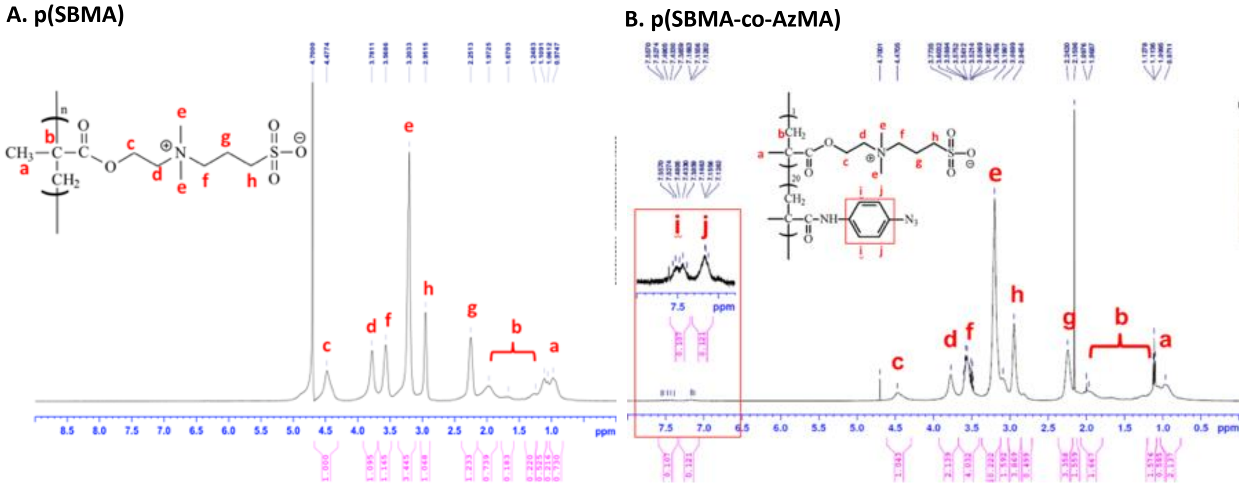

Figure S3 The ^{1}H NMR spectrum of poly(PEGMA-co-AzMA)

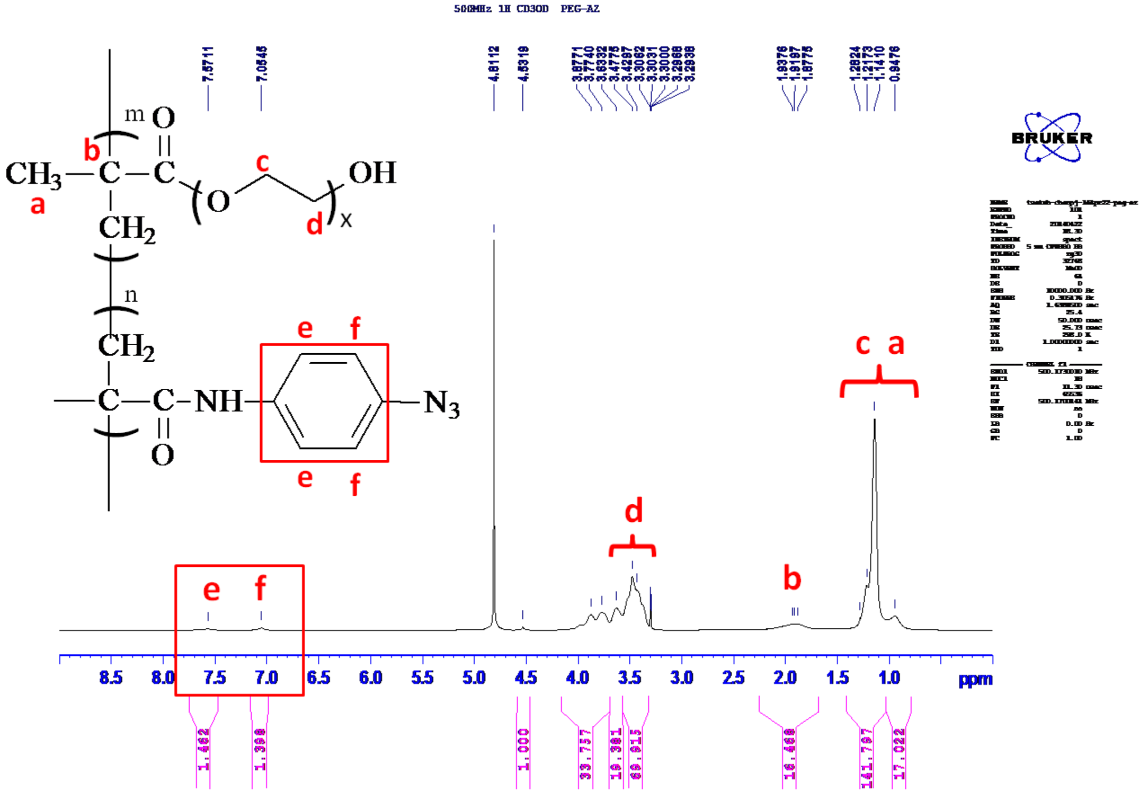

Figure S4 The ^1H NMR spectrum of poly(PNIPAAm-co-Az)

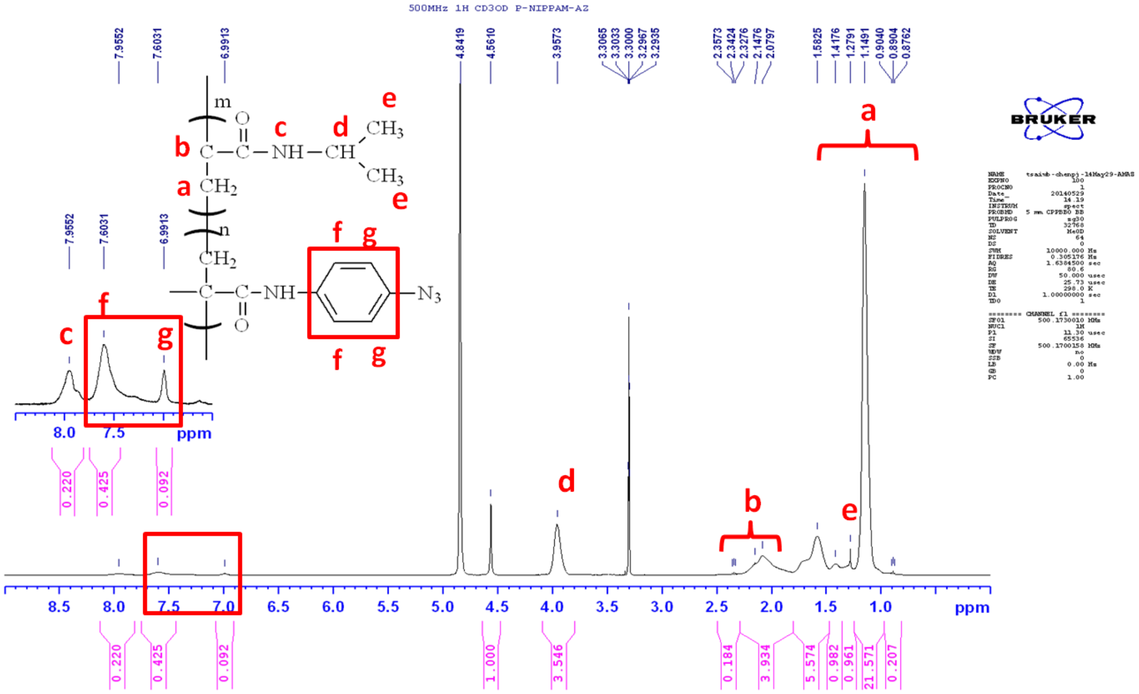

Supplement: Supplementary file 1 [file polymers-14-00225-s001.zip › supplementary.pdf]
